# A snapshot of microbial diversity and function in an undisturbed sugarcane bagasse pile

**DOI:** 10.1186/s12896-020-00609-y

**Published:** 2020-02-28

**Authors:** Leigh Gebbie, Tuan Tu Dam, Rebecca Ainscough, Robin Palfreyman, Li Cao, Mark Harrison, Ian O’Hara, Robert Speight

**Affiliations:** 1 Queensland University of Technology, 2 George St, Brisbane, QLD 4000 Australia; 2 Metabolomics Australia, Australian Institute for Bioengineering and Nanotechnology (AIBN), The University of Queensland, St. Lucia, QLD 4072 Australia

**Keywords:** Sugarcane, Bagasse, Lignocellulose, Biomass, Cellulase, Xylanase, Laccase, Peroxidase, Amplicon sequencing, Fungi

## Abstract

**Background:**

Sugarcane bagasse is a major source of lignocellulosic biomass, yet its economic potential is not fully realised. To add value to bagasse, processing is needed to gain access to the embodied recalcitrant biomaterials. When bagasse is stored in piles in the open for long periods it is colonised by microbes originating from the sugarcane, the soil nearby or spores in the environment. For these microorganisms to proliferate they must digest the bagasse to access carbon for growth. The microbial community in bagasse piles is thus a potential resource for the discovery of useful and novel microbes and industrial enzymes. We used culturing and metabarcoding to understand the diversity of microorganisms found in a uniquely undisturbed bagasse storage pile and screened the cultured organisms for fibre-degrading enzymes.

**Results:**

Samples collected from 60 to 80 cm deep in the bagasse pile showed hemicellulose and partial lignin degradation. One hundred and four microbes were cultured from different layers and included a high proportion of oleaginous yeast and biomass-degrading fungi. Overall, 70, 67, 70 and 57% of the microbes showed carboxy-methyl cellulase, xylanase, laccase and peroxidase activity, respectively. These percentages were higher in microbes selectively cultured from deep layers, with all four activities found for 44% of these organisms. Culturing and amplicon sequencing showed that there was less diversity and therefore more selection in the deeper layers, which were dominated by thermophiles and acid tolerant organisms, compared with the top of pile. Amplicon sequencing indicated that novel fungi were present in the pile.

**Conclusions:**

A combination of culture-dependent and independent methods was successful in exploring the diversity in the bagasse pile. The variety of species that was found and that are known for biomass degradation shows that the bagasse pile was a valuable selective environment for the identification of new microbes and enzymes with biotechnological potential. In particular, lignin-modifying activities have not been reported previously for many of the species that were identified, suggesting future studies are warranted.

## Background

Bagasse is the fibrous material remaining after sugarcane stalks are crushed to remove the sugar and is a major source of lignocellulose. In 2018 for example, 188 million tons of sugar was produced around the world which would have produced approximately 180–200 million tons of bagasse [1].

At most mills the bagasse is used to fuel boilers, co-generating steam and electricity [2]. However it could have various higher value uses: second generation biofuels [3]; fibres for paper, particle board [4] and in 3D printing [5]; xylan-based products such as xylooligosaccharides [6]; substrate for edible or medicinal mushroom growth [7]; substrate for single-cell protein, enzyme, or other high-value microbial products [8, 9]; and high value chemicals from the lignin fraction [10] are some examples. Bagasse has advantages such as low ash content (~ 2.5% compared to 11–14% in other plants, [9]) and as a C4 crop, sugarcane is one of the most efficient converters of energy into biomass. However, the potential for value-adding to bagasse and other lignocellulose feedstocks is primarily limited by the recalcitrant nature of the polymers present and the lack of efficient and sustainable (economic and environmental) conversion processes. Biological processing is one approach and although progress in our understanding of biomass decomposition is continually improving, there is still potential to further exploit the genetic diversity of adapted microbial communities inhabiting environments rich in lignocellulose.

When bagasse leaves the mill it contains approximately 50% water and of the remaining dry matter, 50% cellulose, 25% hemicellulose, 25% lignin, a small percentage of sucrose and very little nitrogen (0.1–0.5%) [11]. Excess bagasse is often stored in large open-air stockpiles. Several studies have shown that these piles become unique environmental niches with distinctive micro-conditions, such as gradients in temperature, pH and oxygen, and diverse microbial populations depending on the position in the pile and/or age of the bagasse [12–16]. Culturing [17–21], metabarcoding, and metagenomics [13, 14, 22–27] on bagasse pile samples or associated soil has revealed a unique microbial community compared to other lignocellulosic environments and the presence of novel biomass-degrading microbes and enzymes. In addition, composting-like conditions are more efficient than submerged fermentation for enriching organisms with cellulases [28] and presumably other carbohydrate hydrolases. Mello et al., also found that a microbial consortium grown under nutrient limiting conditions on sugarcane bagasse, became more diverse and enriched in lignocellulose-degrading enzymes than the same consortium grown on rich media [29]. However, there is still much to learn about bagasse-enriched microbes, especially fungi, because at present the contribution of their lignocellulolytic enzymes to biomass degradation has been underestimated since both their presence and role has not been extensively investigated in relevant metagenomic studies. Indeed, Mhuantong et al., [13] observed that a relatively small proportion of a bagasse fosmid library represented fungal DNA (less than 4.3% of total) but that all of the lytic polysaccharide monooxygenases (CaZy AA9) in the sample were of fungal origin.

Previous bagasse microbe studies have focused on either culturing or extensive sequence-based analyses. There is benefit however to using both approaches [30–32]. While culture-independent methods have come to the fore due to the estimate that approximately 99% of microbes cannot be cultured [33], culturing is still necessary for understanding microbial function, physiology, micro-diversity and community dynamics in the environment [34] and to be able to use the microbes and associated enzymes in industrial processes. Both methods also have biases. Culturing depends on the choice of media and conditions and microbes that are not really living in the pile can be cultured from spores. With PCR-based methods, DNA from non-viable organisms can be amplified and the primers can amplify certain sequences better than others. The DNA extraction step can also bias certain organisms and according to human microbiome studies, for sequencing, microbes should be present at 10^5^ cells/mL whereas they can be cultured from as low as 10^2^ cells/mL [32]. Finally, for both methods but especially culture-independent studies, microbe identification relies on database quality and completeness.

The aim of this study was to explore the diversity of microbes in bagasse and their potential for lignocellulose conversion. In contrast to previous studies, we used both culture dependent and culture-independent methods to describe the fungi and bacteria associated with different positions in a relatively small and uniquely undisturbed Australian bagasse pile. We sampled at different positions because the pile was deposited gradually over several sugarcane milling seasons so that bagasse deeper in the pile had been there longer. However due to the large size of the pile, we were only aiming for a ‘snapshot’ of the organisms present, to explore the potential of this environment, rather than a comprehensive analysis, as this was practically not feasible. In a first step towards characterising their function and potential for novel enzyme activities, we screened the cultured microbes for lignocellulose-degrading enzymes, including lignin-modifying enzymes.

## Results

### Bagasse sampling and analysis

**Fig. 1:**
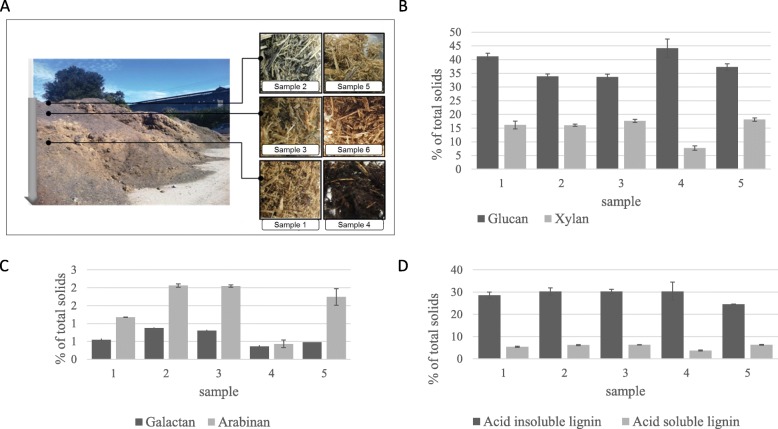
Bagasse sampling and compositional analysis. **a** Photos of the Rocky Point bagasse pile and samples taken at different positions in the pile. **b** Glucan and xylan as % of total solids in five different bagasse samples. Glucan was significantly higher (*p* < 0.05) in Samples1 and 4 compared to others and xylan was significantly lower (*p* < 0.05) in Sample 4 compared to others. **c** Galactan and arabinan as % of total solids in five different bagasse samples. Both were significantly lower in Samples 1 and 4 compared to others (*p* < 0.01). **d** Acid insoluble and soluble lignin as % of total solids in five different bagasse samples. Acid soluble lignin was significantly lower in Samples 1 and 4 (*p* < 0.05). Error is standard deviation and *n* = 3

Seven samples were collected in different positions from the bagasse stockpile at the Rocky Point mill in Queensland Australia as detailed in Fig. 1a and Table 1. The temperature ranged from approximately 50 °C (Table 1) in the deeper layers that we sampled to 30 °C on the surface. The pH was low in the first deep layer sample we collected but the deep layer was of similar pH to the other layers 6 months later (Table 1), suggesting temporal and environmental variability at equivalent depths across the pile. The chemical compositional analysis showed notable changes in the deep samples and especially the oldest and deepest sample (Sample 4) compared to other samples. Sample 1, and to a further extent, Sample 4 also showed visual signs of modification and degradation (Fig. 1a). The sugars issuing from hemicellulose, arabinan and galactan were significantly lower (*p* < 0.001) in Samples 1, 4 and 5, and xylan, was also significantly lower (*p* < 0.05) in Sample 4 than in the other samples (Fig. 1b, c). Acid soluble lignin contents were also significantly lower (*p* < 0.05) in the deep samples compared to the other samples (Fig. 1d). These differences could be due to microbial activity combined with the low pH of the pile leading to degradation. Glucan, on the other hand, was proportionally higher in both samples from the deeper layers suggesting that cellulose or the glucan component of hemicellulose was less degraded than the C5 components of hemicellulose.

**Table 1:** Characteristics of bagasse samples from the Rocky Point sugarcane mill

	Sample 1	Sample 2	Sample 3	Sample 4	Sample 5	Sample 6	Sample 7
Date	May 2016	May 2016	May 2016	February 2017	February 2017	February 2017	February 2017
Depth (cm)	60	Top crust	10	80	10	30	Top crust^a^
In situ Temperature (°C)	50	30	30	46	28	38	39
pH	3	5	5.5	5	4.85	4.8	ND
Water (%)	55	47	75	80	67	77	ND
Analysis	Culturing;16S amplicon sequencing	Culturing; ITS and 16S amplicon sequencing	Culturing; ITS and 16S amplicon sequencing	Culturing; ITS and 16S amplicon sequencing	ITS and 16S amplicon sequencing	ITS and 16S amplicon sequencing	Culturing

^a^ visible fungal growth

### Oil-producing yeast, biomass-degrading fungi, *Bacillus* and *Streptomyces* cultured from the bagasse

In total, 104 microbes were cultured from bagasse samples collected at the Rocky Point sugarcane mill in May 2016 and February 2017. The strains and how they were selected are summarised in Additional file 1. 16S or ITS sequences were used to query the 16S ribosomal sequence (bacterial and archaeal) database at NCBI or the UNITE [35] database, respectively. The top BLAST hit based on e-values was noted even though in some cases the sequence matched several sequences in the database with the same percentage identity.

The microbes were isolated in two separate rounds of culturing. The samples were rinsed to remove spores on the surface and the samples were ground in Tween detergent to isolate organisms strongly adhered to the bagasse. In the first round, fresh samples were incubated on rich media and isolates compared between three samples from the top (Sample 2), 10 cm under the crust (Sample 3) and 60 cm deep (Sample 1), with a focus on yeast and filamentous fungi. Indeed, *Bacillus* dominated plates without chloramphenicol and these were the only bacteria isolated besides one *Burkholderia* species (RP31) which was resistant to chloramphenicol. Only four *Bacillus* isolates (*B. amyloliquefaciens* and *B. megaterium*; nominated as RP1, RP2, RP3 and RP5) and one fungal species, closest to *Talaromyces flavus* (RP4) were cultured from the deep sample (1). From the top of the pile, yeast from six different genera and filamentous fungi from seven different genera were cultured. Four yeast and six fungi were cultured from the 10 cm sample (Additional file 1).

Next, selective plating was carried out with the aim of isolating thermophilic and mesophilic biomass-degrading enzyme producing organisms. For this, new bagasse samples were obtained from 80 cm deep (Sample 4), which as mentioned above appeared to be substantially degraded, and we also cultured a sample from the surface with obvious fungal growth (Sample 7). Forty-eight microorganisms including bacteria (10), yeast (14) and filamentous fungi (24) were cultured from the 80 cm sample. Apart from *Bacilli*, the only bacteria isolated were *Streptomyces* (*Streptomyces mexicanus* and *clavus*, RP52, RP53, RP64 and RP81), with *S. mexicanus* seemingly outcompeting or inhibiting all other microorganism when grown on carboxy-methyl cellulose at 50 °C. It was also the only isolate that grew alongside *Thielavia* species on xylan at 50 °C. *Thielavia* species also grew on rich media at 50 °C as well as on cellulose and xylan as the sole carbon sources. However, when bagasse was included in the media and the plates were incubated at 50 °C, the fast-growing *Aspergillus fumigatus* outgrew any other organism. At 28 °C, the *Aspergillus* was outcompeted by *Talaromyces*. Similarly, at the lower temperature, yeast outgrew *Streptomyces* and filamentous fungi on Azo-xylan media. Twelve fungi from the top of the pile grew on lignin-containing media at 28 °C.

In the selective culturing some isolates of the same species were cultured under more than one condition (Additional file 1). For example, strains most similar to *Thielavia terrestris* (RP40, 41, 43, 44, 45, 54, 76, 77, 78, 80, 81, 83) were cultured at 50 °C, on CMC, xylan and guaiacol. While most of the partial 16S or ITS sequences of the cultured strains were 99–100% identical to a sequence of a known species in the databases, some showed slightly less similarity (Additional file 1). The rRNA sequence of one isolated organism (RP12) showed similarity to a sequence only identified in the databases as *Ascomycota sp.* but its morphology more resembled *Coniochaeta/Lecythophora.* Two other *Coniochaeta* strains (RP62 and RP68) also appeared to be quite different from those with sequences in the databases and their partial rRNA sequences were also deposited at NCBI. Two strains whose morphology resembled *Rhizopus* (RP38 and RP94) and one with similarity to *Mucoromycotina* (RP34) could not be sequenced with these primer sets (Additional file 1).

Many of the yeast isolates cultured from the bagasse are reported to accumulate high levels of microbial oil, for example *Rhodoturola*/*Rhodosporidium* (RP7, 8, 14, 15, 28, 58, 66, 67 and 69) [36, 37], *Cryptococcus*/*Papiliotrema laurentii* (RP13, 27, 29, 61, 65 and 70) [38] and *Meyerozyma caribbica* (RP6, 16 and 30) [39] as well as other closely related species such as *Naganishia* (RP60).

### Enzyme screening of bagasse microbes

**Fig. 2:**
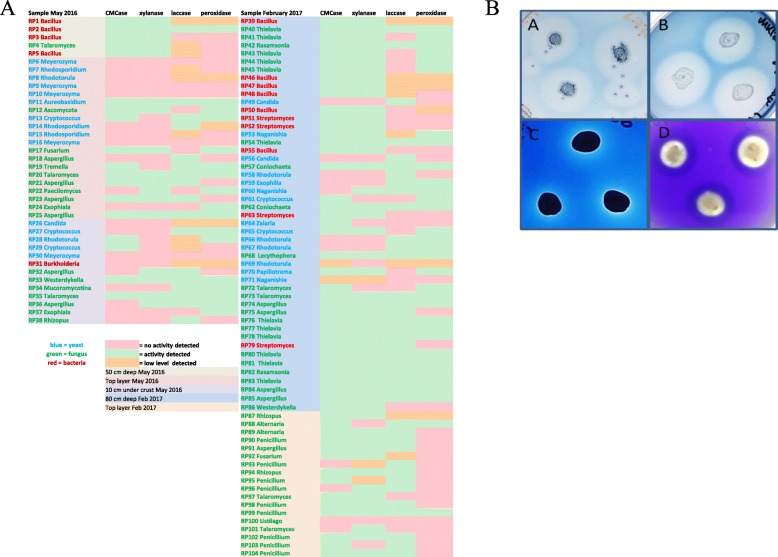
Screening of bagasse microbes for biomass-degrading enzymes. **a** The bacteria, yeast and filamentous fungi (RP1-RP103) isolated from different positions in the pile were screened for cellulase, xylanase, laccase and peroxidase on carboxy-methyl cellulose with trypan blue dye, azo-xylan, remazol brilliant blue and azure B containing agar plates, respectively. Activity was scored as positive or not based on clearance zones around the colonies. Low activity indicates a very minor clearance zone that took longer than 7 days to become visible. Examples of each assay are shown in **b**

All 104 isolates were screened in semi-quantitative assays on solid media for the production of biomass-degrading enzymes (Fig. 2b). The results are summarised in Fig. 2a and an example of each assay is shown in Fig. 2b. Overall, 70, 67, 70 and 57% of the isolated microorganisms showed CMCase, xylanase, laccase and peroxidase activity, respectively and approximately 29% displayed all four activities. There was little difference in the proportion of fungi from the top or 10 cm deep layers that produced biomass-degrading enzymes. For example, 38 and 31% of the fungi isolated from Sample 2 and 3 showing xylanase activity. However, 100% (5 microorganisms) from Sample 1 produced CMCase and xylanase and in Sample 4, xylanase activity was found for 80% of the microorganisms, CMCase for 85%, laccase for 67% and peroxidase for 73% of organisms. All four activities were found for 44% of Sample 4 organisms.

**Fig. 3:**
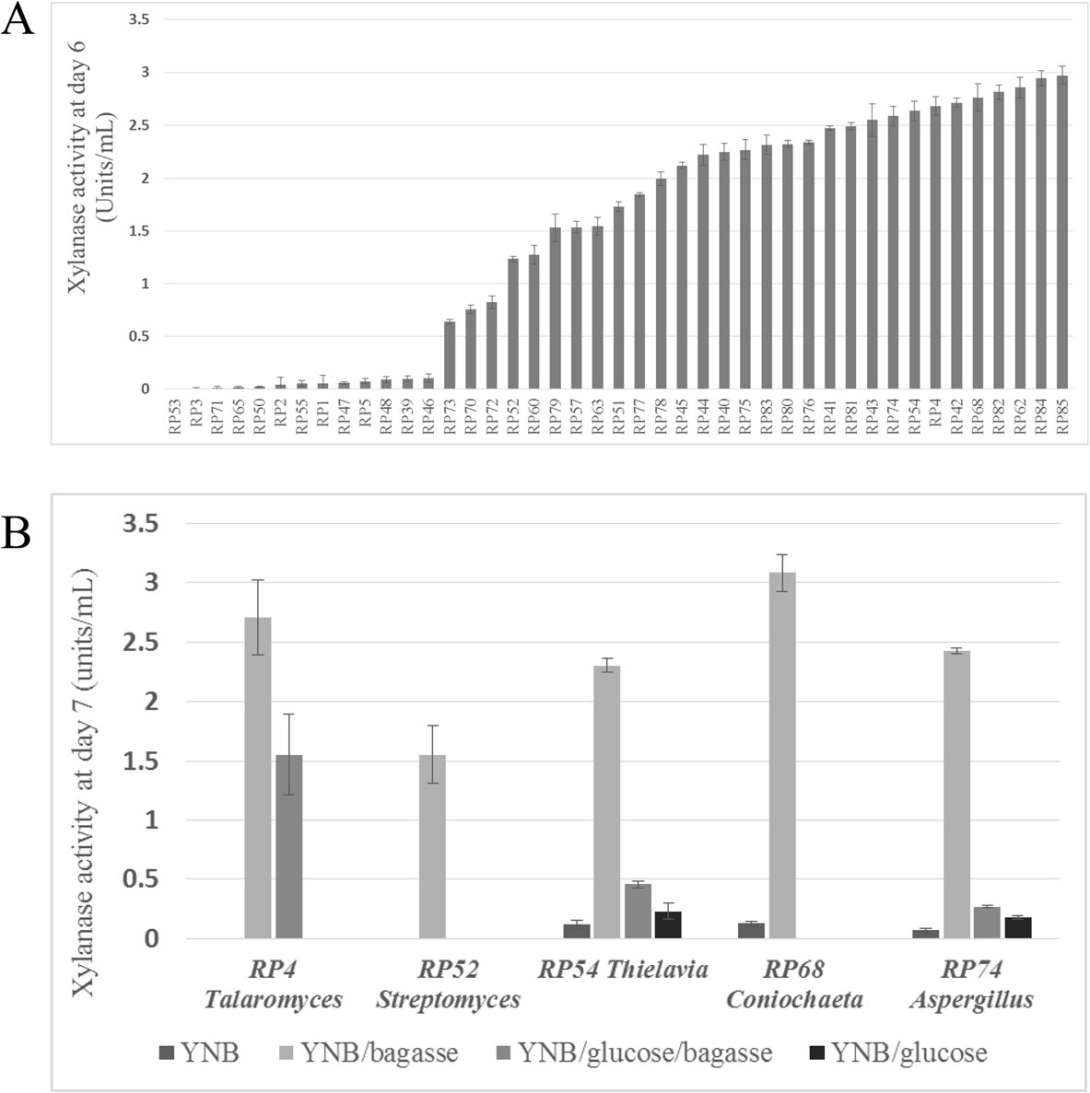
Bagasse induces xylanase activity among the isolates. **a** Xylanase activity was measured for all the strains isolated from the deep layers (Samples 1 and 4) that showed activity in the plate assay. The assay was carried out on strains grown in 24 well plates for six days in yeast nitrogen base (YNB) media containing 2% sugarcane bagasse as the carbon source. **b** Xylanase activity of a selection of the strains in YNB media with and without bagasse and glucose after seven days, showing that the bagasse induced xylanase. Xylanase activity is expressed as units per ml. Error is standard deviation and *N* = 3 in the assay

To confirm the functional presence of these organisms in the bagasse pile, we grew the 44 strains that were positive on xylan agar plates from the deep layers (Samples 1 and 4) in minimal media containing bagasse as the sole carbon source and measured xylanase activity. Almost all the strains showed some level of activity, which approximately correlated with the level of activity on agar plates (based on the size of the clearance zone and speed of clearance), validating the screening approach (Fig. 3a). The highest activities were shown by *Aspergillus terreus* (RP84/85) [40], *Rasamsonia emersonii* (RP42/82), *Aspergillus fumigatus* (RP74/75), *Thielavia terrestris* and the as yet uncharacterised xylanase producers *Talaromyces rugulosus* (RP4), *Coniochaeta taeniospora* (RP62/68) [41] and *Streptomyces mexicanus* (RP51) [42]. *Westerdykella dispersa* (RP86) was the only definite ‘false-positive’ with obvious growth but no xylanase activity even after 7 days in bagasse media. To confirm that the bagasse was inducing the activity, several strains were grown in media with and without bagasse or glucose and activity was the highest with bagasse as the only carbon source (Fig. 3b).

### Amplicon sequencing-overview

Amplicon sequencing was used to rapidly gain insight into the diversity of microbes present in the bagasse pile, especially those that were not cultured. With16S primers, a total of 608,084 three-hundred base pair pair-end reads were obtained with approximately 56,000 to 120,000 reads per sample. A total of 347,222 reads were obtained from ITS primer sequencing, ranging from approximately 55,000 (Sample 3) to 88,000 reads for Sample 2. Presumably due to the low microbial load in the initial deep layer sample (Sample 1) as shown by culturing, no products were amplified with the ITS primers. The number of reads amplified with 16S primers from this sample was also much lower than for the other samples (approximately 56,000 versus 100,000 to 120,000 for others). The reads were submitted to the NCBI Short Read Archive (SRA) under BioProject ID PRJNA530327 according to the minimum information about any (x) sequence (MIxS) specifications [43].

**Fig. 4:**
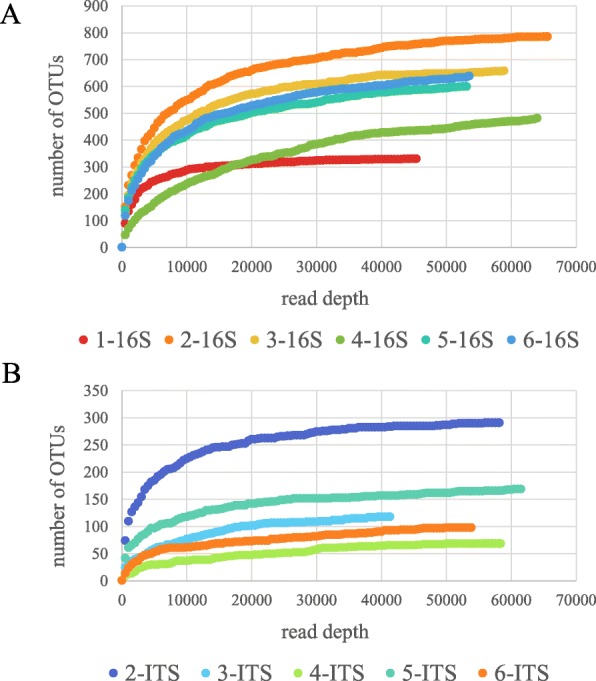
Rarefaction curves for 16S and ITS amplicon sequences for each sample. **a** Rarefaction curve for 16S amplicons. Species richness was highest in Sample 2 and lowest in Samples 1 and 4. **b** Rarefaction curve for ITS amplicons. Species richness was highest in Sample 2 and lowest in Sample 4

In all the samples, 1747 different bacterial and 363 fungal sequences were amplified (Additional files 2 and 3). The number of operational taxonomic units (OTUs) for each sample ranged from 332 (Sample 1, deep layer) to 787 (Sample 2, top layer) for 16S sequencing and 67 (Sample 4, deep layer) to 285 (Sample 2, top layer) for ITS, reflecting, as shown in Figs. 4 and 7, that there was more diversity in the top than deeper layers of the bagasse pile.

**Fig. 5:**
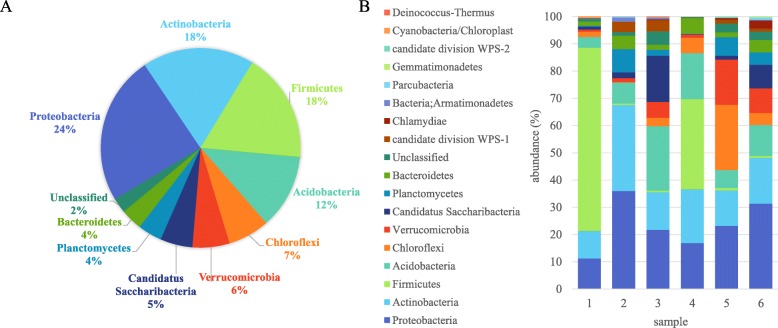
Proportion of major bacterial phyla found in the bagasse samples according to amplicon sequencing. **a** Overall classification of 16S amplicons assigned to operational taxonomic units at the phylum level from all samples. **b** Classification of 16S amplicons for each sample

Taxonomic classification (Additional files 4 and 5) showed overall that 19 bacterial phyla were found in the bagasse pile with *Proteobacteria* (~ 23%), *Actinobacteria* (~ 17%), *Firmicutes* (~ 17%) and *Acidobacteria* (~ 11%) dominating (Fig. 5a).

**Fig. 6:**
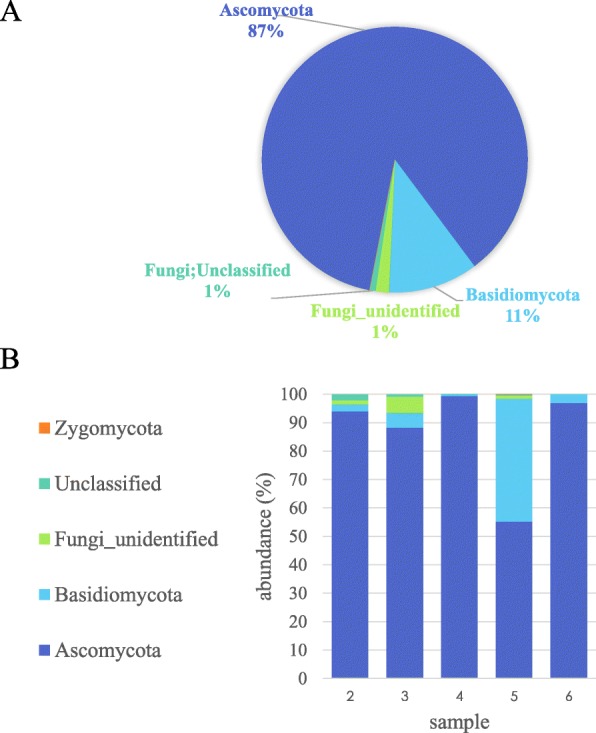
Proportion of fungal phyla found in the bagasse samples according to amplicon sequencing. **a** Overall classification of ITS amplicons assigned to operational taxonomic units at the phylum level from all samples. **b** Classification of ITS amplicons for each sample

Approximately 87% of the fungi identified were *Ascomycota* and 10% *Basidiomycota*, with a small proportion of *Zygomycota* and unidentified/unclassified fungi (Fig. 6a). The *Basidiomyces* were mostly found in Sample 5 (30 cm depth) (Fig. 6b).

At the class level, 45% of the total reads were unclassified *Ascomycota* (Additional file 5). However, among the classified reads, the *Eurotiomycetes* (32% of total; for e.g. *Rasamsonia*, *Talaromyces*, *Aspergillus*) dominated across all samples followed by the *Agaricomycetes* (10% of total) which accounted for almost all the *Basidiomycota*. Lastly, *Dothideomycetes* were found to represent about 5% of the total reads and were mostly in Sample 2 (top layer).

### Bacterial diversity in the pile – dominance of thermophiles and acidophiles

**Fig. 7:**
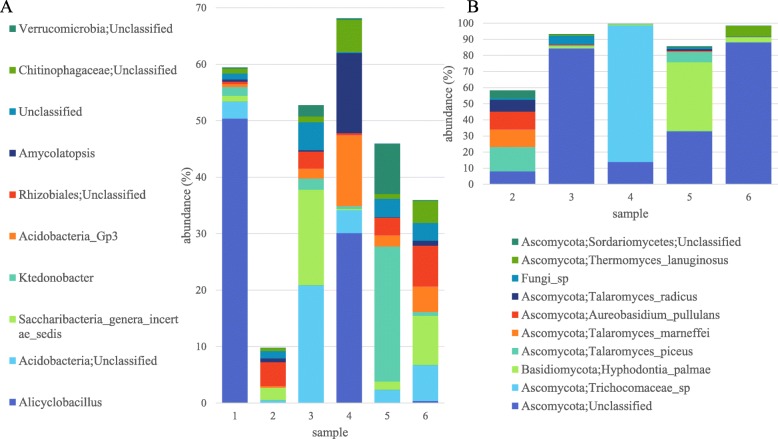
Major bacterial genera and fungal species found in the bagasse samples according to amplicon sequencing. **a** Top 10 most abundant bacteria at the genus level showing their abundance in each sample. **b** Top 10 most abundant fungi at the species level showing their abundance in each sample

OTUs were classified to the genus level for bacteria (Fig. 7a; Additional file 4). The highest total number of bacterial reads, due to their abundance in the two deep layer samples (1 and 4), were *Alicyclobacillus*. This thermo- and acid-tolerant genus was also found in Thai bagasse [11], has well characterised biomass degrading capacity, particularly of xylan [44, 45] and also thrives on both pentose and hexose sugars [46]. However, it has been more commonly associated with fruit juice contamination rather than any other environment. At the species level, the most abundant reads were 99% identical to the newly identified species *Alicyclobacillus kakegawensis* [47] and 98% identical to 16S of the type strain *Alicyclobacillus acidocaldarius* [48]. Overall, 12 different *Alicyclobacillus* sequences were amplified from the samples (Additional file 2).

An unclassified *Acidobacteria* was also very abundant in the deep samples and samples under the crust but not in the top samples (Fig. 7a). The sequence of the representative read for this classification was 100% identical to that of another newly identified thermotolerant species, *Acidobacterium ailaaui*, from a geothermally heated Hawaiian microbial mat [49]. This strain can grow from pH 4.5 to 6, at 15 °C to 55 °C and can metabolise xylose and arabinose. Finally, the third most abundant bacteria, found mostly in Sample 2 (top) belongs to the *Chloflexi* phyla and is 92% identical to the *Ktedonobacteria* (Fig. 5a) *Thermosporothrix narukonensis* and *Thermosporothrix hazakensis,* which are both thermophilic and able to hydrolyse cellulose and xylan [50, 51].

In terms of other bacteria in the pile that may be producing biomass-degrading enzymes, in a previous study [13] all the major phyla in the pile were producing hemicellulose and cellulose degrading enzymes but lignin-modifying enzymes were mostly being produced by *Bacteroidetes* and *Proteobacteria*. However, in the present study *Bacteroidetes* were not as abundant as *Firmicutes* and *Actinobacteria* and in Sample 4 the lignin degrading bacteria *Amycolatpsis* [52] was abundant. We also found a relatively high abundance of *Sulfobacillus* from the *Clostridia* family in our deep layer samples. The other most abundant bacteria sequenced are shown in Fig. 7a and listed in Additional files 2 and 4.

### Fungal diversity in the pile –ubiquitous biomass degraders and novel organisms

ITS OTUs were classified using the UNITE database. The most abundant fungi found in all five samples, with highest levels in Samples 3 and 6, were unclassified *Ascomycota* (Fig. 7b), represented by 54 different sequences (Additional file 3). Overall, 138 of the 370 unique amplicons correspond to uncharacterised fungi in UNITE and NCBI. Thirteen of these sequences, classified as unidentified fungi (UNITE SH230936.06FU/SH189980.06FU/SH211973.06FU) appear to be ciliated protozoa rather than fungi. Among the other 125, some (for example DENOVO1 in Additional file 3) had sequence homology with uncultured fungi from environmental samples but others could be individually classified with the most up-to-date version of UNITE (see below).

The second most abundant fungus, which was initially classified as unidentified *Trichocomaceae sp*. (Fig. 7b), was identified as *Rasamsonia emersonii* (previously *Talaromyces emersonii*) on more detailed individual analysis. This fungus was also cultured from the deep layer. *R. emersonii* is a well characterised moderately thermophilic fungus which produces thermostable xylanase and cellulase as well as other enzymes [53–56]. The strain isolated here produced all four enzymes and was a relatively high xylanase producer. Laccase and peroxidase production are reported for the first time here for this species.

The next most common fungal sequences amplified was for *Basiomycota Hyphodontia palmae/dispersa* from Sample 5 (accounting for the high *Basiomycota* reads from this sample). To date, this fungus is not well characterised.

Another abundant fungus among the sequences was *Talaromyces piceus,* found from the top of the pile at both sampling dates. Two other *Talaromyces* were also among the top hits (*T. marneffei* and T. *radicus*). The culturing experiments isolated the highly related *T. ruber*, *T. amestolkiae*, *T. rugulosus*, *T. funiculosus* and *T. flavus*, which all have very similar ITS sequences and appearances and are also anamorphs (or were renamed) to various *Penicillium* species [57]. While, *T. piceus* was recently shown to possess a novel lignocellulolytic enzyme system [58], *T. amestolkiae* produces diverse β-glucosidases [59, 60], and *T. funiculosus* (also known as *P. funiculosum*) is used commercially to degrade xylan in animal feed [61], the biomass degrading capability of the other isolates has not been reported until now.

The important industrial dimorphic yeast, *Aureobasidium pullulans* was sequenced and cultured from the top layer and produced all four enzymes [62–64]. Another well-known hemicellulose degrader *Thermomyces lanuginosus* [65] was also identified primarily in Sample 6 by sequencing. DNA from the white rot fungi *Phanerochaete chrysosporium* [66], which was never cultured, was amplified from samples from the top of the pile but not from the deeper samples.

Among the unknown *Ascomycota* sequences, the most abundant in Sample 4 was used to query the latest UNITE database and identified as *Thielavia terrestris* (accession KU729090), which, as mentioned above, was the major fungus cultured from this sample. Amplicon sequencing also showed that it was prevalent in samples closer to the top of the pile but not on the surface (DENOVO4 in Additional file 3). Most of the fungi that were cultured from Samples 1, 2, 3, 4 and 7 such as *Aspergillus terreus, Aspergillus fumigatus*, *Alternaria*, *Fusarium*, *Coniochaeta*, *Rhodotorula*, *Exophilia*, and *Cryptococcus* were represented in the amplicons at least at the genus level. We did not amplify *Rhizopus* or *Paecilomyces*, a genus of the phylum *Deuteromycota*, which as mentioned above were cultured and have been previously associated with sugarcane bagasse.

## Discussion

In this study we assessed bagasse microbiota using a novel approach of both culturing and amplicon sequencing in combination with enzyme screening. In general, industrial bagasse piles are influenced by complex environmental factors. This pile was unique as it was relatively small and static over 9 months due to mill shutdown and thus had time for microbial communities and chemistries to develop. Normally, bagasse piles are transient, and bagasse is added or removed frequently. They are built up in uneven layers and each layer is also likely to have experienced different microbial loads during harvesting, processing, and deposition, and different conditions (rain or wind, dirty machinery, etc.) during deposition on the pile. Regardless, this study shows the potential of sugarcane bagasse piles for isolating novel biomass-degrading microbes and discovering new biomass-modifying enzymes. There may be the potential to develop methods to replicate, in the lab or field, the conditions which led to the bagasse colonisation and degradation and thus enrich for adapted microbes.

One hundred and four microbes were isolated from the bagasse pile in two separate rounds of culturing. The first non-selective round focused mostly on fungi and a second selective round aimed to specifically isolate biomass degraders. The strains isolated included *Bacillus* and *Streptomyces* bacterial species, a diverse range of yeast and known biomass-degrading filamentous fungi. Many of the strains belonged to the same species even though they showed different morphotypes on the same or different media. In the selective culturing some isolates of the same species were cultured under more than one condition (Additional file 1). Overall, the dynamics of populations observed under the different cultivation conditions used shows the importance of experimental design for such experiments and the relevance of our parallel genomic experiments to observe total populations.

The strains were named based on the high similarity of their rRNA sequences with known sequences in the databases, but high sequence conservation at the rRNA level does not preclude that the isolates may have evolved unique phenotypes/genotypes in response to environmental stimuli.

Many of the yeast isolates cultured from the bagasse such as *Rhodoturola*/*Rhodosporidium* [36, 37], *Cryptococcus*/*Papiliotrema laurentii* [38] and *Meyerozyma caribbica* [39] are reported to accumulate high levels of microbial oil. Indeed we demonstrated that the bagasse-derived strain RP15 (*Rhodosporidium toruloides*) produced higher yields of intracellular microbial oil when grown on pre-treated bagasse compared to synthetic media and compared to the ATCC type strain [67]. These yeast can grow with extremely low nitrogen concentrations [68], which may explain their prevalence in the nitrogen deficient bagasse pile, with low nitrogen media also used to induce microbial oil production. These, as well as other non-oleaginous yeast isolated, such as *Candida*, *Aureobasidium*, *Coniochaeta/Lecythophora* and *Exophilia*, may also be associated with sugarcane or soil in sugarcane plantations as many of the same species were also isolated from leaves and stems sampled close to the soil or from bagasse [13, 21].

Some of the other microbes isolated were also cultured previously from sugarcane and bagasse, such as *Aspergillus fumigatus* and *Aspergillus niger* (*tubingensis*), *Paecilomyces*, *Tremella*, *Rasamsonia emersonii* and others [12, 15, 17, 20, 21]. *Thielavia terrestris* is an efficient thermophilic biomass-degrader and a strain was previously isolated from Brazilian bagasse [19, 69]. *Bacillus subtilis* was prevalent in liquid samples collected at sugarcane mills with the authors proposing that it lives on sugarcane [70].

Amplicon sequencing gave rapid insight into the diversity of microbes present in the bagasse pile, especially those that were not cultured. Microbial diversity decreased in the deeper layers of the pile presumably due to increased specialisation. This decreased diversity was seen as variation in the distribution of bacteria and fungi from different phyla between samples (Figs. 5b and 6b). For example, *Firmicutes* dominated the deeper samples but were much less prevalent in top samples. These bacterial phyla also dominated in deeper layers of other piles under potentially similar environmental conditions (wet season) [14, 22]. However under potentially drier conditions (dry season) and in less degraded bagasse, *Acidobacteria* dominated [24].

There was less diversity among the fungi sequences that were amplified. However, ITS primer sequences for metabarcoding environmental DNA are still under development [71] and although the primers used here were selected based on their reported ability to amplify both *Ascomycota* and *Basidiomycota* [72, 73] primer specificity could have biased the DNA that was amplified [74].

The most abundant bacteria in the pile, *Alicyclobacillus, Acidobacteria* and *Ktedonobacteria* species are known thermophiles and acidophiles [46, 49, 51] showing adaptation to the conditions in the pile. The bacteria that we cultured from the bagasse pile, *Bacillus*, *Streptomyces* and *Burkholderia* were sequenced in all the samples but at relatively lower abundance. *Bacillus* reads were mostly amplified from Sample 1 (Additional file 2) but were still cultured from Sample 4. The differences in abundance between culturing and sequencing results also shows the benefit of performing both analyses in the same study.

The most abundant fungal sequences appeared to correspond to currently unidentified fungi, suggesting that novel fungi were present in the pile. However, the other highly abundant fungal sequences that were amplified were related to the thermophiles cultured in this study (*R. ermersonii* and *T. terrestris* as well as *Thermomyces lanuginosus*)*.* In the only other culture-independent study of fungi in bagasse, Rattanachomsri et al., [14] sequenced 24 *Ascomycota*, including some that we also found, such as *R. ermersonii* and *T. lanuginosaus*. ITS amplicon sequencing of DNA extracted from a maize straw compost pile with added nitrogen found that *T. lanuginosaus* dominated the fungal population but there was also some *Aspergillus* and *Talaromyces*, as found here, among a few other genera [75].

The isolated organisms were screened for common lignocellulose degrading enzymes (cellulase, xylanase, laccase, peroxidase) and a large majority (up to 85%) of the strains expressed at least one enzyme, with 29 and 44% of organisms from the non-selective and selective rounds of culturing respectively, showing all four activities. The high proportions of microorganisms displaying hydrolase activity was also likely related to the selective enrichment in culturing for these activities. Overall, the microbes that had colonised the deeper parts of the bagasse pile where biomass modification was observed were mostly functionally specialised for lignocellulose decomposition and our targeted approach successfully enriched microorganisms with biomass degrading potential.

Under the conditions used here, especially in the first round of culturing, we mostly cultivated fast growing species. Other strategies such as plating on very dilute media or high throughput dilution plating could be used to isolate slower growing oligotrophs, but whether these would be better enzyme producers remains to be seen. Indeed, when such an approach was taken by Shrestha et al. [18], only 8 out of the 106 fungi they isolated from *Miscanthus* and sugarcane trash, could deconstruct *Miscanthus* cell walls, although they did perform better than the most widely used fungus *Trichoderma reesei* [34]. For industrial applications, fast growing organisms, as obtained here, with high-titre enzyme production are generally favourable.

The yeast from the non-selective culturing were the least likely to be producing the enzymes. For example, the *Meyerozyma*, which was cultured five times showed none of the four enzyme activities. However, an isolate from this species was previously shown to have exoglucanase activity, which we did not test for here [76]. In the targeted approach however, the yeast isolates mostly expressed one or two enzymes. To our knowledge, some of these species of potentially oleaginous yeasts such as those belonging to the extremophile genus *Naganishia* [77, 78] have never been shown to produce carbohydrate hydrolases before. An early study of microbes growing in bagasse [15] found similarities in the microbial composition of naturally stored versus fermented bagasse with a succession of organisms growing in the bagasse coinciding with their enzyme activity. Yeast first dominated as they consumed the residual sucrose, then bacteria took over and degraded hemicellulose and cellulose, finally the other fungi infiltrated, digesting the hemicellulose, cellulose and lignin. In this study, yeasts were only found in the top layers of the pile at first but some, such as *Rhodoturola*, progressed deeper into the pile with time and with increasing degradation. Modern mills such as the one sampled in this study do not leave as much sucrose in the bagasse as would have been the case in the early study. The yeast here are more likely to be feeding off pentose and hexose sugars released from degradation or using enzymes themselves as has also been observed in rotting wood [79].

Selection of organisms on media containing lignin as the sole carbon source was not effective in selecting microbes with lignin peroxidase activity, with only one strain out of 12 showing strong decolourisation of Azure B. However, most produced laccase, with 5 out of 12 also producing the other two enzymes (xylanase and CMCase) and another 5/12 showing two activities. Azure B decolourisation was initially shown to be specific for lignin peroxidase [41] and is not decolourised by laccase. However, we found yeast and *Bacillus* species with Azure B decolourisation activity, suggesting that other enzymes such as quinone dehydrogenase, as previously shown in *Bacillus* [42, 43], or that some other novel enzyme activity may have been involved. The fungi may also have been able to grow by consuming residual hemicellulose and/or cellulose associated with the lignin and/or by producing agarase. Indeed, many of the microbes cultivated did show some agarase activity (results not shown). Similarly, only one oxidase-producing organism, similar to *Penicillium glabrum* (RP93), was isolated on plates containing tannic acid because all the other colonies that grew were producing tannase (results not shown).

We confirmed the functional presence of these organisms in the bagasse pile by showing that their xylanase production was specifically induced in the presence of bagasse (Fig. 3b). The strains RP4, and RP68, similar to the uncharacterised species *Talaromyces rugulosus* and *Coniochaeta taeniospora,* respectively, showed relatively high xylanase activity, comparable to that of the well-characterised *A. fumigatus* [80, 81] and *T. terrestris* [19, 69] (Fig. 3b). Overall, the three isolated *Coniochaeta* species were among the best enzyme producers in this study, producing relatively high levels of all four enzymes. Recent draft genome sequences of several of these wood rot fungi have revealed their large arsenal of genes for biomass-degradation, including genes encoding novel lignin-degrading enzymes [82, 83]. The cultured strains are now a resource for mining new lignocellulosic modifying enzymes. Finally, the prevalence of biomass degrading microbes in the pile and potentially novel fungi, as shown through amplicon sequencing, suggests that bagasse piles could be a relevant environment for functional metagenomics studies to isolate novel enzymes.

## Conclusions

The culture-dependent approach used here allowed novel biomass-degrading microbes to be isolated. While the glucanases and hemicellulases of many organisms similar to those found here have been well characterised, for many microorganisms the discovery of laccase and peroxidase activity is novel and provides a basis for further study. The culture-independent approach using amplicon sequencing provided data on the overall microbial biodiversity. Good consistency was observed between the fungi that were cultured and the fungi identified in the sequencing although 33% of the *Ascomycota* reads were similar to uncultured or unknown species. The findings from both culturing and amplicon sequencing suggest that relatives of the thermophilic biomass-degrading fungi *T. terrrestris* and *R. emersonii* were the most abundant in the deep layers of the pile and could have been significantly involved in the hemicellulose degradation observed. In terms of bacteria involved in the degradation, the *Firmicutes*, *Bacillus* and *Alicyclobacillus* species may have played a significant role based on their abundance in culturing and amplicon sequencing. Overall, the variety of species that were found in this study and that are known for biomass degradation shows that bagasse piles are a valuable selective environment for the identification of new microbes and enzymes with biotechnological potential.

## Methods

### Bagasse sampling and analysis

Bagasse was sampled from a stockpile at the Rocky Point sugarcane mill in Woongoolba (27.7413°S, 153.3148°E, 7 m AMSL), Queensland, Australia. In 2015, 2016 and 2017 the mill crushed 383,832, 110,231 (smaller than usual amount due to technical difficulties) and 388,484 t, respectively, in the crushing season from July to December [19, 20]. The area has a humid subtropical climate with an average maximum temperature of 26 °C, minimum of 15 °C and an average rainfall of 1087 mm [21] mostly falling from November to March.

Bagasse samples were taken from a stockpile (excess bagasse once most of it was used to run the furnace) at the Rocky Point mill. It is a relatively small pile compared to industry standards, approximately 5 m high and 20 m across. On the 31st May 2016, approximately 6 months since the end of crushing the previous year, samples were taken at three places in the pile: the top, approximately 10 cm under the crust, and approximately 50 cm into the pile. On the 22nd February 2017, further samples were taken on the surface, just below the surface, approximately 30 cm deep and approximately 80 cm deep (Table 1).

For sampling, bagasse was scooped into sterile 50 mL tubes without touching it. Larger samples for analysis were taken with plastic bags. Samples were stored on ice while transported back to the lab. Samples for metagenomics were then stored at − 40 °C, while samples for culturing and analysis were stored at 4 °C and used within days.

The temperature was measured in situ using an Infrared Non-Contact Digital Thermometer. The pH was measured on fresh bagasse using one-part bagasse to 2.5 parts water with a pH meter. The moisture content was determined by oven drying and weighing starting with three replicates of approximately 50 g (wet weight) of sample. For compositional analysis, the bagasse was dried at 40 °C for 3 days and then ground to a fine powder with a Retsch SM 100 cutting mill. To determine the proportions of structural carbohydrates and lignin in the different bagasse samples, 300 mg +/− 10 mg was analysed according to Sluiter et al. [22]. A reference bagasse sample was analysed at the same time. A one-way analysis of variance (ANOVA) was performed on the data using SigmaPlot (version 13.0) to check for significant differences between the samples at *p* < 0.05. All data passed Normality (Shapiro-Wilk) and Equal Variance tests (Brown-Forsythe) tests. Pairwise multiple comparisons were carried you with the Holm-Sidak method. Degrees of freedom was 14.

### Media and plate enzyme assays

Microbes were routinely cultivated on Yeast Peptone Dextrose media (YPD: 2% bacteriological peptone (Oxoid, Thermo Fisher Scientific, Australia); 1% yeast extract (Sigma-Aldrich, Australia); 2% D-glucose (Sigma-Aldrich, Australia); 2% bacteriological agar (Oxoid, Thermo Fisher Scientific, Australia)), Nutrient Agar (Oxoid, Thermo Fisher Scientific, Australia) or Potato Dextrose Agar (PDA; Oxoid, Thermo Fisher Scientific, Australia). Chloramphenicol (Sigma-Aldrich, Australia) was added to plates at 25 mg/mL. For enzyme assays, 1 x (0.68%) Yeast Nitrogen base with amino acids (Sigma-Aldrich, Australia) was used as the base media to which various substrates were added: 0.02% azo-xylan (Azo-wheat arabinoxylan (1% w/v unbuffered) (Megazyme, Australia); 0.5% carboxy-methylcellulose (CMC (Sigma-Aldrich, Australia) with 0.01% Trypan Blue (Sigma-Aldrich, Australia); 0.05% remazol brilliant blue (Sigma-Aldrich, Australia); 0.01% azure B (Sigma-Aldrich, Australia); 0.02% (1.8 mM) Guiaicol (Sigma-Aldrich, Australia); 0.5% lignin (prepared ‘in house’); 2% finely ground bagasse with or without 2% glucose and with 2% bacteriological agar. For xylanase assays, media was made with 1x YNB and 2% finally ground bagasse with or without glucose.

To observe whether microbes in the collection have cellulase (endoglucanase-CMCase), xylanase, laccase or lignin peroxidase activity, they were grown on media containing substrate and/or dyes described in media section. These substrates were validated using purified enzymes (e.g. Accellerase 1500 (Genencor)) and /or organisms known to show the activity (for e.g. *Botrytis* for laccase) when possible. Yeast or bacteria were patched in a quarter or third of a plate whereas fungi were patched in three replicates on a single plate to avoid cross-contamination. The plates were incubated for 7 days at 28 °C or 50 °C and clearing zones and growth observed. No attempt was made to quantify the activity, a positive activity was noted for any clearance zone produced within the 7 days. However, if it took longer than 7 days for the clearance zone to appear, the activity was noted as low.

### Xylanase assay of microbes growing in bagasse containing media

The production of xylose, cleaved from beechwood xylan, was quantified using the dinitrosalicylic acid reducing sugar (DNS) assay [23], adapted for microplates. 50 μL of diluted enzyme in DNS assay buffer (100 mM Acetate buffer pH 5.0, 20 mM CaCl2, 0.01% Tween 20) was pipetted into 200 μL wells of a 96-well PCR plate containing 125 μL of 0.4% (weight/volume) beechwood xylan, mixed and incubated for 30 min at 37 °C. After incubation, 75 μL of DNS stop reagent was added to each sample and samples were heated to 100 °C for 5 min before the absorbance at 530 nm was measured. Each enzyme dilution was quantified in triplicate. For blanks, the substrate and DNS stop reagent were mixed first, followed by the addition of the dilute enzyme sample. One unit of enzyme activity was defined as the amount of sample that released 1 μmol of reducing sugar equivalents from xylan substrate per minute under the assay conditions used.

### Culturing and identification

To culture microbes (bacteria and fungi) from the bagasse, approximately 1 g of bagasse was ground in 10 mL of NaCl/ Tween80 (0.9%/0.01%) and then mixed for 30 min. Serial dilutions were then plated out and incubated at 28 °C or 50 °C. Distinct morphotypes were selected and restreaked to obtain pure cultures which were then stored as glycerol stocks (30% glycerol for yeast or bacteria or 12.4% glycerol plus 0.04% tween-80 for fungal spores). Microbes were identified by sequencing barcode regions. For this, DNA was extracted using the Lithium Acetate/SDS protocol of Looke et al. [24]. Then 1 μL was used to amplify the full length 16S, or partial 18S or ITS sequence from bacteria, yeast or filamentous fungi respectively using the primers shown in Table 2.

**Table 2:** primers used for rRNA amplification and sequencing

Microbe	Primer sequencing	References	OneTaq annealing temp (°C)	Approximate Product size (bp)
Bacteria	27F 5′-AGAGTTTGATCCTGGCTCAG-3′ 1492R 5′-ACGGCTACCTTGTTACGACT-3’	[84]	52	1.5 kb
Fungi (including yeast)	ITS1F 5’-TCCGTAGGTGAACCTGCGG-3′ ITS4R 5′-TCCTCCGCTTATTGATATGC-3’	[85]	49	600 bp
Fungi (but not yeast)	SCTB6-F 5’-GCATATCAATAAGCGGAGG-3′ LR3-R 5′-CCGTGTTTCAAGACGGG-3’	[21]	48	550 bp
yeast	NL1F 5’-GCATATCAATAAGCGGAGGAAAAG-3′ NL4R 5′-GGTCCGTGTTTCAAGACGG-3’	[86]	51	550 bp

PCR was carried out using OneTaq (NEB) following the manufacturer’s instructions and the annealing temperature shown in Table 2 for 35 cycles. Products were sequenced at the Central Analytical Research Facility at the Queensland University of Technology. Sequences were used to query the 16S ribosomal sequence (bacterial and archeae) database at NCBI or the UNITE [35] database, respectively. The strains were identified to the species level (when possible) based on the top BLAST hit (e-values) and % identity.

### Amplicon sequencing

Microbial genomic DNA was extracted from approximately 250 mg of bagasse using the DNAeasy Powersoil Kit including PowerBead tubes (Qiagen Australia) following the manufacturer’s instructions. For sample 1 (50 cm from May), more than one extraction was carried out and pooled and precipitated in an attempt to obtain sufficient DNA. PCRs were then performed on 5–10 ng of this gDNA using primers targeting the V3 and V4 region [87] or the ITS2 region for fungi [26]. Illumina adapter overhang nucleotide sequences were added to the gene-specific sequences. The full-length primer sequences (using standard IUPAC nucleotide nomenclature) were:

16S Amplicon PCR Forward Primer 5’-TCGTCGGCAGCGTCAGATGTGTATAAGAGACAGCCTACGGGNGGCWGCAG-3′

16S Amplicon PCR Reverse Primer -

5′-GTCTCGTGGGCTCGGAGATGTGTATAAGAGACAGGACTACHVGGGTATCTAATCC-3′ ITS3_KYO2 5′-TCGTCGGCAGCGTCAGATGTGTATAAGAGACAGGATGAAGAACGYAGYRAA-3′

ITS4_KYO3 5′-GTCTCGTGGGCTCGGAGATGTGTATAAGAGACAGCTBTTVCCKCTTCACTCG-3′

Amplifications were carried out with Q5 polymerase (New England Biolabs, Genesearch Australia) following the manufacturer’s instructions using 1 μM primer and at five different annealing temperatures: 53 °C, 55 °C, 58 °C, 60 °C, 63 °C for 16S and 54 °C, 58 °C, 60 °C, 62.5 °C and 65 °C for ITS for 25 cycles. Large primer dimers (~ 150 bp) obtained with the ITS primers were removed by cutting pooled PCR fragments out of a gel and purifying the DNA with ISOLATE II PCR and Gel Kit (Bioline, Meridian Bioscience, Australia) following the manufacturer’s instructions. Amplicons were prepared for Illumina sequencing using the MiSeq Reagent Nano Kit v2 (Illumina Australia) and the MiSeq Reagent Kit v3 (600-cycle) following the manufacturer’s instructions. Pooled indexed libraries were then sequenced on an Illumina MiSeq to yield 300 bp paired end sequences (version 3 technology).

### Metabarcoding sequencing analysis

Amplicon sequences were processed and assigned to operational taxonomic units (OTUs) using the MICrobial Community Analysis (Micca) pipeline [88] (version 1.6.2) using the docker image and following the paired-end sequencing tutorial: https://micca.readthedocs.io/en/latest/pairedend_97.html. Briefly, paired end reads were merged, trimmed to remove primer sequences and then quality filtered. To characterize the taxonomic structure of the samples, the sequences were then organized into Operational Taxonomic Units (OTUs) at 97% identity using the ‘otu’ command which implements de novo greedy clustering. OTUs were then classified using the Ribosomal Database Project II classifier (version 11.5) for 16S sequences [28] and the UNITE database (version 7.2) for ITS sequences [29]. Finally, Micca was used to summarize and rarefy the data to compare the taxonomic composition of each sample. Sampling heterogeneity was reduced by rarefaction to a read depth of 45,000 and 41,000 for 16S and ITS, respectively. The full list of commands used are shown in Additional file 6.

## Supplementary information

**Additional file 1.** List of the strains isolated from bagasse including a detailed description of isolation conditions and homology of rRNA sequences.

**Additional file 2.** List of unique 16S amplicons assigned to individual OTUs, their taxonomic classification to the genus level and the number of times these were amplified from each sample (after rarefaction to 45,000 to reduce sample heterogeneity).

**Additional file 3.** List of unique ITS amplicons assigned to individual OTUs, their taxonomic classification to the species level and the number of times these were amplified from each sample (after rarefaction to 41,000 to reduce sample heterogeneity).

**Additional file 4.** Taxonomic classification of 16S OTUs at six levels (kingdom to genus) and their abundance in each sample (after rarefaction).

**Additional file 5.** Taxonomic classification of 16S OTUs at seven levels (kingdom to species) and their abundance in each sample (after rarefaction).

**Additional file 6.** List of commands used to run Micca for 16S and ITS analysis.

## Data Availability

The datasets generated and/or analysed during the current study are available as follows: The strains are described in Additional file 1 and are stored at QUT at the authors’ address. Partial ribosomal RNA sequences for the three *Coniochaeta* isolates were submitted to NCBI under the following accession numbers: MN216224 (RP12), MN218196 (RP62), MN218197 (RP68). The 16S and ITS reads were deposited at the NCBI short read archive under BioProject ID: PRJNA530327 https://www.ncbi.nlm.nih.gov/Traces/study/?acc=PRJNA530327 Taxonomic classification of the amplicon sequencing data is provided in Additional files 2, 3, 4 and 5.

## Abbreviations

CMC: Carboxy-methyl cellulose
DNS: Dinitrosalicylic acid reducing sugar
gDNA: Genomic DNA
ITS: Internal transcriber sequence
NCBI: The National Centre for Biotechnology Information
OTU: Operational Taxonomic Units
PCR: Polymerase chain reaction
