# Supplementary material for: A snapshot of microbial diversity and function in an undisturbed sugarcane bagasse pile
Source: BMC Biotechnol. 2020 Feb 28;20:12. doi: 10.1186/s12896-020-00609-y (PMC7049217; doi:10.1186/s12896-020-00609-y)
Supplement: Supplementary file 6 — Additional file 6. List of commands used to run Micca for 16S and ITS analysis. [file 12896_2020_609_MOESM6_ESM.pdf]

Additional file 6

Micca commands (version 1.6.2)

16S

micca mergepairs -i reads16S/*_R1*.fastq -o reads16S/merged.fastq

micca stats -i reads16S/merged.fastq -o reads16S/merged.stats

micca trim -i reads16S/merged.fastq -o reads16S/trimmed.fastq -w CCTACGGGNGGCWGCAG -r GACTACNVGGGTWTCTAATCC -W -R -c

micca filterstats -i reads16S/trimmed.fastq -o reads16S/trimmed.stats

micca filter -i reads16S/trimmed.fastq -o reads16S/filtered.fasta -e 0.5 -m 400

micca otu -m denovo_greedy -i reads16S/filtered.fasta -o reads16S/denovo_greedy_otus -t 4 -c

micca classify -m rdp -i reads16S/denovo_greedy_otus/otus.fasta -o reads16S/denovo_greedy_otus/taxa.txt

micca msa -m nast -i reads16S/denovo_greedy_otus/otus.fasta -o reads16S/denovo_greedy_otus/msa.fasta --nast-template core_set_aligned.fasta.imputed --nast-threads 8

micca tree -i reads16S/denovo_greedy_otus/msa.fasta -o reads16S/denovo_greedy_otus/tree.tree

micca root -i reads16S/denovo_greedy_otus/tree.tree -o reads16S/denovo_greedy_otus/tree_rooted.tree

micca tobiom -i reads16S/denovo_greedy_otus/otutable.txt -o reads16S/denovo_greedy_otus/tables.biom -t reads16S/denovo_greedy_otus/taxa.txt

micca tablestats -i reads16S/denovo_greedy_otus/otutable.txt -o reads16S/denovo_greedy_otus/tablestats

micca tablerare -i reads16S/denovo_greedy_otus/otutable.txt -o reads16S/denovo_greedy_otus/otutable_rare.txt -d 45000

micca tabletotax -i reads16S/denovo_greedy_otus/otutable.txt -t reads16S/denovo_greedy_otus/taxa.txt -o reads16S/denovo_greedy_otus/taxtables

micca tabletotax -i reads16S/denovo_greedy_otus/otutable_rare.txt -t reads16S/denovo_greedy_otus/taxa.txt -o reads16S/denovo_greedy_otus/taxtables_rare

micca tablebar -i reads16S/denovo_greedy_otus/taxtables_rare/taxtable$i.txt -o reads16S/denovo_greedy_otus/taxtables_rare/taxtable$i.png

micca tablebar -i reads16S/denovo_greedy_otus/otutable.txt -o reads16S/denovo_greedy_otus/otutable_plot.png

ITS

micca mergepairs -i readsITS/*_R1*.fastq -o readsITS/merged.fastq

micca stats -i readsITS/merged.fastq -o readsITS/merged.stats

micca trim -i readsITS/merged.fastq -o readsITS/trimmed.fastq -w GATGAAGAACGYAGYRAA -r CTBTTVCCKCTTCACTCG -W -R -c

micca filterstats -i readsITS/trimmed.fastq -o readsITS/trimmed.stats

micca filter -i readsITS/trimmed.fastq -o readsITS/filtered.fasta -e 0.5 -m 335

micca otu -m denovo_greedy -i readsITS/filtered.fasta -o readsITS/denovo_greedy_otus -t 4 -c

micca classify -m rdp --rdp-gene fungalits_unite -i readsITS/denovo_greedy_otus/otus.fasta -o readsITS/denovo_greedy_otus/taxa.txt

micca msa -m nast -i readsITS/denovo_greedy_otus/otus.fasta -o readsITS/denovo_greedy_otus/msa.fasta --nast-template core_set_aligned.fasta.imputed --nast-threads 8

micca msa -i readsITS/denovo_greedy_otus/otus.fasta -o readsITS/denovo_greedy_otus/msa.fasta --nast-threads 8

micca tree -i readsITS/denovo_greedy_otus/msa.fasta -o readsITS/denovo_greedy_otus/tree.tree

micca root -i readsITS/denovo_greedy_otus/tree.tree -o readsITS/denovo_greedy_otus/tree_rooted.tree

micca tobiom -i readsITS/denovo_greedy_otus/otutable.txt -o readsITS/denovo_greedy_otus/tables.biom -t readsITS/denovo_greedy_otus/taxa.txt

micca tablestats -i readsITS/denovo_greedy_otus/otutable.txt -o readsITS/denovo_greedy_otus/tablestats

micca tablerare -i readsITS/denovo_greedy_otus/otutable.txt -o readsITS/denovo_greedy_otus/otutable_rare.txt -d 41000

micca tabletotax -i readsITS/denovo_greedy_otus/otutable.txt -t readsITS/denovo_greedy_otus/taxa.txt -o readsITS/denovo_greedy_otus/taxtables

micca tabletotax -i readsITS/denovo_greedy_otus/otutable_rare.txt -t readsITS/denovo_greedy_otus/taxa.txt -o readsITS/denovo_greedy_otus/taxtables_rare

micca tablebar -i readsITS/denovo_greedy_otus/taxtables_rare/taxtable$i.txt -o readsITS/denovo_greedy_otus/taxtables_rare/taxtable$i.png

micca tablebar -i readsITS/denovo_greedy_otus/otutable.txt -o readsITS/denovo_greedy_otus/otutable_plot.png
